# Anal Sphincter Disruption in Open Pelvic Fractures: A Case Report Highlighting the Importance of Early Intervention

**DOI:** 10.7759/cureus.78186

**Published:** 2025-01-29

**Authors:** Rafique Umer Harvitkar, Alfredo Tonsi, Hussein Al-Najjar, Ioannis Hannadjas, Mariyam Shaheed

**Affiliations:** 1 General Surgery, Royal Sussex County Hospital, Brighton, GBR; 2 Gastrointestinal Surgery, Royal Sussex County Hospital, Brighton, GBR

**Keywords:** anal sphincter injury, diverting colostomy, pelvic fracture, perineal injury, sepsis

## Abstract

Anal sphincter injuries, often accompanying perineal trauma, are closely associated with pelvic fractures (PFs). If unrecognized and untreated, these injuries can lead to sepsis and fecal incontinence. We present the case of a 25-year-old male patient who sustained severe trauma, resulting in unstable open PFs and associated perineal injuries, including anal sphincter damage. The patient was managed with an early diverting colostomy to prevent pelvic sepsis, followed by staged reconstruction of the pelvic ring. This case underscores the importance of a systematic and thorough approach to managing sphincter injuries in post-trauma care. Clinicians must maintain a high index of suspicion for perineal injuries in patients with open PFs, as early intervention can significantly improve functional outcomes.

## Introduction

Anal sphincter injuries, although often underrecognized, are a significant complication of pelvic trauma, particularly in cases involving open pelvic fractures (PFs). These injuries, which can lead to long-term functional impairments such as fecal incontinence, present a major challenge in the management of trauma patients [1-3]. The pelvic region’s complex anatomy, with its proximity to vital structures such as the rectum, bladder, and reproductive organs, makes it particularly vulnerable to damage during high-velocity trauma, such as road traffic accidents. Approximately 10% of PFs are unstable and typically result from such high-velocity impacts. The mortality rate for PFs is significantly elevated (14%) in cases of missed or incorrect diagnosis and treatment. Perineal lacerations, which occur in about 2%-4% of PFs, can lead to anal sphincter injuries in a significant proportion of cases. Studies suggest that approximately 11%-14% of PFs with perineal lacerations result in anal sphincter damage [4-6].

When PFs are associated with perineal lacerations, the risk of anal sphincter injury increases, although such injuries may not always be immediately apparent. While the overall incidence of anal sphincter injury in pelvic trauma is relatively low (around 2%-6%), even minor disruptions to the sphincter mechanism can result in severe, long-term consequences. These injuries are frequently compounded by other pelvic organ injuries, including damage to the bladder, urethra, and rectum, requiring a multidisciplinary approach to treatment. Early diagnosis and prompt intervention are crucial in preventing long-term morbidity. Timely surgical repair, along with measures to control hemorrhage and prevent infection, can significantly improve functional outcomes and reduce the risk of permanent incontinence [3-6].

This paper aims to review the pathophysiology, diagnosis, and management strategies for anal sphincter injuries following pelvic trauma, emphasizing the importance of early recognition and appropriate surgical intervention to optimize patient outcomes.

## Case presentation

A 24-year-old male motorcyclist who was involved in a road traffic accident was brought to the regional trauma center. Upon arrival at the Accident and Emergency Department, he presented with hemorrhagic shock (class II), with a blood pressure of 110/60 mmHg and a heart rate of 108 beats/minute. His Glasgow Coma Scale score was 15/15. Initial investigations revealed a base deficit of 8.1 mmol/L and a lactate level of 5.8 mmol/L. The major hemorrhage protocol was promptly activated.

The primary survey identified injuries to the chest, pelvis, and abdomen, along with a large, deep perineal laceration. The patient responded well to initial resuscitation, with an improvement in tachycardia to 97 beats/minute. Blood tests showed a hemoglobin level of 88 g/L, a hematocrit of 0.40 L/L, and a white blood cell count of 25 × 10^9^/L. Urinalysis results were normal.

**Figure 1:**
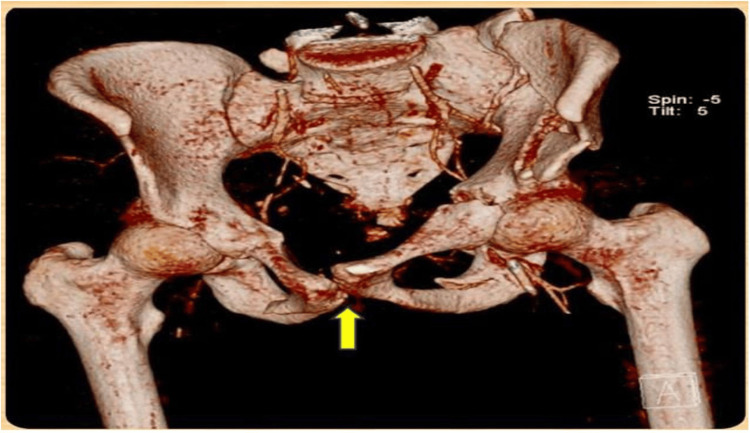
3D CT scan of PF with displacement. The yellow arrow indicates the fracture site PF: pelvic fracture

A pelvic binder and perineal wound packing were applied. Given the severity of his injuries, a full trauma CT scan of the head, chest, abdomen, and pelvis was performed. The CT findings include the following: an open PF was noted in the pelvic region (Figure 1); surgical emphysema and bilateral pneumothorax were observed in the thoracic region; and grade 3 spleen and grade 2 kidney lacerations, along with an intra-abdominal hematoma, were observed in the abdomen.

Immediate fluid resuscitation was initiated, and a multidisciplinary team was assembled, including specialists in orthopedics, vascular intervention, urology, general surgery, and plastic surgery. Due to persistent hemorrhagic shock, the patient was promptly taken to the operating theater for damage control laparotomy.

Step 1 involves performing a laparotomy along with initial surgical management, specifically a damage control laparotomy. Under general anesthesia, the patient underwent a splenectomy and control of abdominal hemorrhage. A bilateral chest tube was placed for pneumothorax management. Step 2 includes the second look surgery after three days in the intensive therapy unit. The patient underwent a second-look surgery, which included perianal repair.

**Figure 2:**
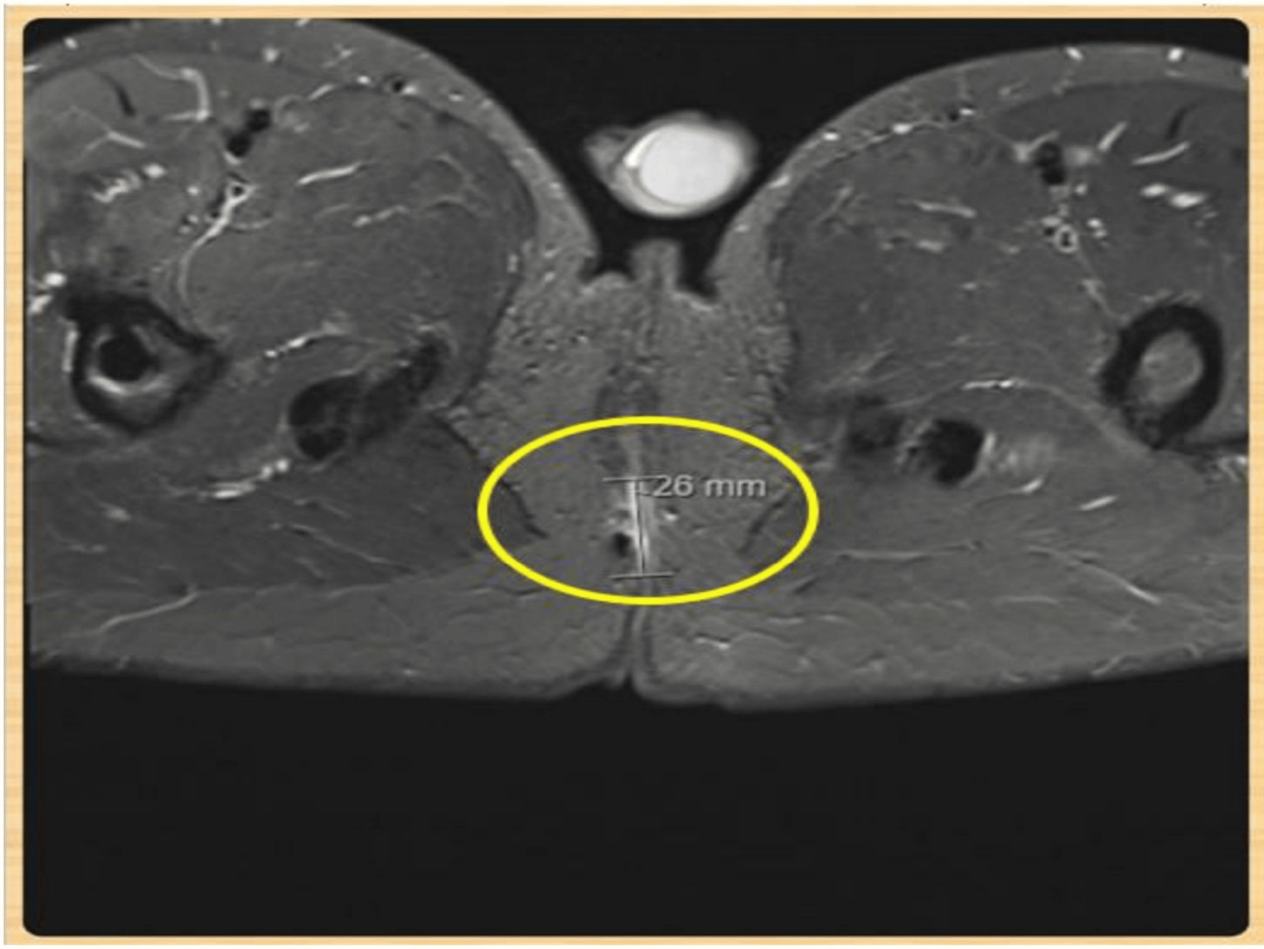
Pelvic MRI showing a 26-mm perineal injury accompanied by associated soft tissue disruption

**Figure 3:**
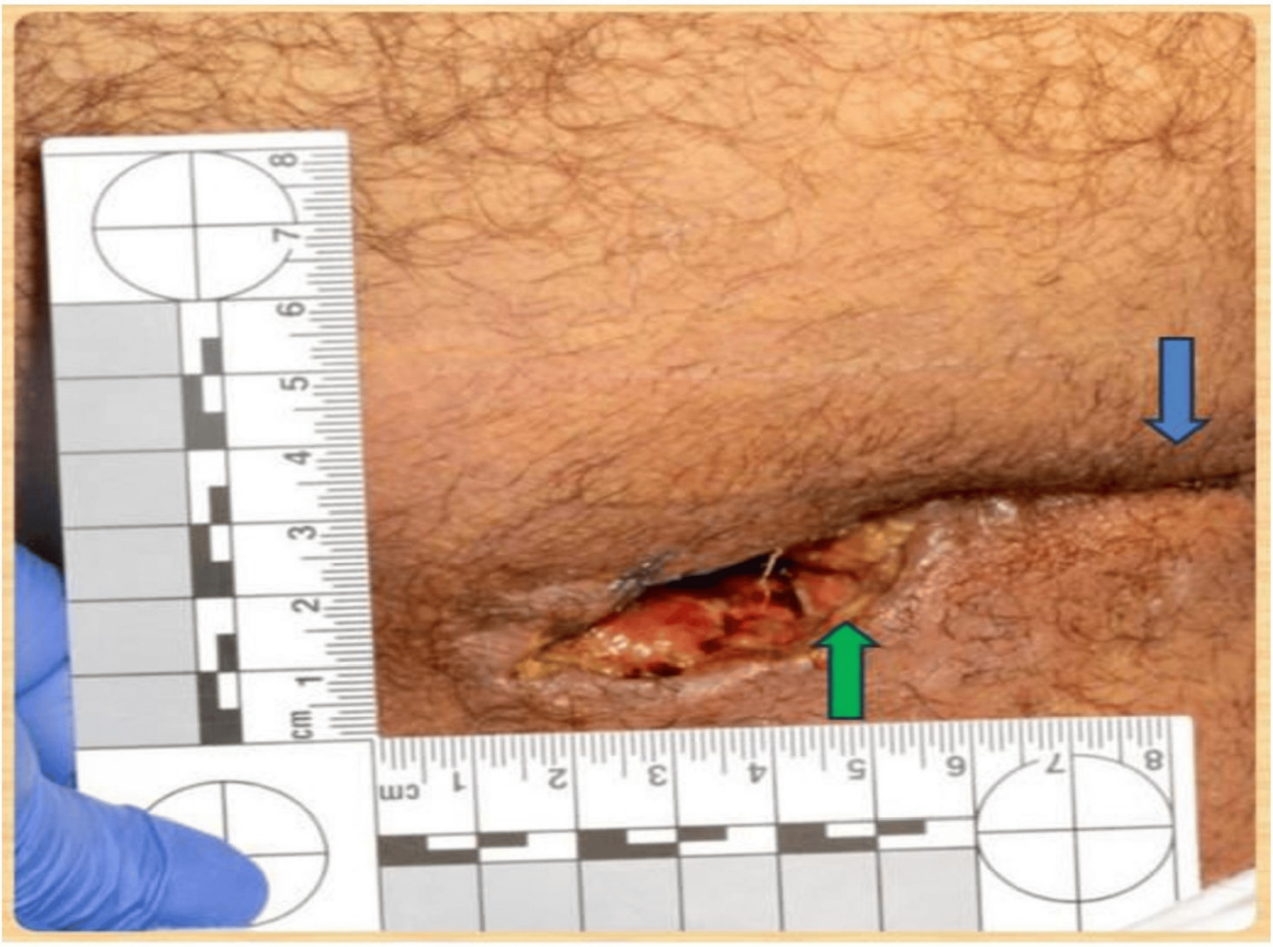
Perineal laceration with measurement markings following trauma. The green arrow indicates laceration, and the blue arrow indicates repair with Prolene

The perineal wound exploration findings are shown in Figure 2: superficial laceration measuring 6 cm in length at the urogenital perineum, extending 1 cm above the anal verge at the 12 o’clock position; deep posterior perineal laceration measuring 8 cm in depth, resulting in a complete disruption of the external anal sphincter and a muscular defect involving the posterior pelvic floor; external anal sphincter injury, completely transected between the 5 and 7 o’clock positions (Figure 3); pelvic floor muscular defect, which involves the pubococcygeus and iliococcygeus muscles located between the 6 and 9 o’clock positions; and rectal laceration that is small and superficial, and located at the 7 o’clock position.

Procedure

The wound was debrided and irrigated in the anterior perineum. Superficial laceration was noted in the intact anterior puborectalis muscle. The posterior perianal wound was inspected thoroughly. Pelvic floor repair was performed by closing the right-sided pelvic floor defect using 2-0 Vicryl (Ethicon Inc., Bridgewater, NJ). External sphincter repair was done by repairing the injury between the 5 and 7 o’clock positions using an overlapping technique with 3-0 Vicryl. The wound was closed with 2-0 Prolene (PROLENE™ 2-0, Ethicon Inc., Bridgewater, NJ) after thorough irrigation (Figure 3).

Rigid sigmoidoscopy up to 13 cm showed normal mucosa without tears or bleeding. The perineal wound was irrigated with saline using a jet wash. Active bleeding from the lacerated area was controlled, and the wound was closed in layers with careful repair of the external anal sphincter. A drain was placed, and a temporary diverting colostomy was performed to prevent pelvic sepsis.

Step 3 includes pelvic reduction and external fixation. Six months after the injury, the external fixator was removed. A flexible sigmoidoscopy, conducted three months after surgery before colostomy closure, showed nearly healed superficial and deep wounds, with normal resting and squeezing pressures in the anal canal. Mild defunctioning colitis was observed. The patient subsequently underwent a successful stoma reversal without complications. The patient was also assessed via phone consultation, during which anal function was evaluated using tools such as the Vaizey incontinence test (the score was zero).

## Discussion

The detection and management of anal sphincter injuries in pelvic trauma present unique challenges, particularly in the context of high-energy mechanisms and complex soft tissue damage. Early identification during the secondary survey or following damage control surgery is critical, as immediate repair of these injuries has yielded significantly better long-term functional outcomes, especially in young patients [7].

Early identification of anal sphincter injuries

Anal sphincter injuries are frequently underdiagnosed during the initial trauma management phases. This can be attributed to the overshadowing of life-threatening injuries and the subtle presentation of sphincter disruptions amidst extensive pelvic trauma. Early intervention for anal sphincter injuries is generally defined as surgical repair performed within the initial 24-72 hours following trauma, ideally during damage control or secondary procedures. A systematic approach to perineal and anorectal assessment is essential during secondary surgery or reexploration following damage control interventions. Key clinical indicators, such as extensive perineal lacerations, displaced anal anatomy, or anal avulsion, should prompt further evaluation [7,8].

Direct intraoperative inspection remains the most reliable method for detecting these injuries in trauma settings. Adjunct imaging modalities, such as endoanal ultrasound or MRI, may provide additional diagnostic precision in selected cases, but the emergent nature of these scenarios often limits their application. Establishing a high index of suspicion and incorporating a focused anorectal evaluation as a routine component of pelvic trauma management can improve early detection rates [9,10].

Benefits of immediate sphincter repair

Immediate repair of anal sphincter injuries offers distinct advantages over delayed approaches. In young patients, early intervention mitigates the risk of chronic dysfunction, fibrosis, and muscle atrophy, all of which are more pronounced in delayed repairs. Restoring sphincter continuity at the earliest opportunity promotes better long-term functional outcomes, including continence preservation and improved quality of life [11,12].

Emerging evidence supports the role of early intervention in reducing complications such as fecal incontinence and the psychological burden of prolonged sphincter dysfunction. Immediate repair also aligns with broader principles of trauma care, emphasizing the restoration of anatomy and function as soon as the patient’s condition permits. Studies have demonstrated that delayed repair is associated with higher rates of incontinence, particularly in younger patients, whose recovery potential might otherwise be maximized with early intervention [7].

Challenges in immediate repair despite its benefits

Immediate repair of anal sphincter injuries is not without challenges. Hemodynamic instability, contamination from associated injuries, and significant soft tissue loss may necessitate deferral of definitive repair. In such cases, identifying sphincter injuries during initial or damage control procedures ensures that repair can be prioritized in subsequent surgeries. Additionally, meticulous debridement and surgical precision are crucial to minimize infection risks and optimize outcomes [3,13].

To improve outcomes in pelvic trauma involving anal sphincter injuries, we recommend incorporating thorough perineal and anorectal evaluations into secondary surveys and during reexploration following damage control surgery.

In managing sphincter injuries, immediate repair should be prioritized whenever feasible, particularly in young patients, to optimize functional recovery and enhance quality of life. Given the complex nature of such injuries, a multidisciplinary approach involving colorectal and trauma surgeons is essential to ensure comprehensive management and improved patient outcomes.

Moreover, developing protocols emphasizing early detection and standardized repair methods, guided by input from colorectal experts, is crucial for achieving better long-term functional results.

## Conclusions

The early identification and immediate repair of anal sphincter injuries in pelvic trauma are pivotal in achieving favorable long-term outcomes. This is particularly critical in younger patients, where functional preservation has a profound impact on quality of life. Integrating systematic evaluation techniques and prioritizing sphincter repair by pelvic floor specialists during secondary surgeries or re-explorations can help bridge the gap between injury detection and optimal recovery. Further research and standardized pathways protocols are needed to refine these practices and improve care for this unique patient population.
